# Data analysis for SEM-EDX, thermokinetics, surfactant, and corrosion inhibition activity of Co(II) and Zn(II) complexes of pyrrole-based surfactant ligand

**DOI:** 10.1016/j.dib.2023.109124

**Published:** 2023-04-07

**Authors:** Janak Adhikari, Ajaya Bhattarai, Narendra Kumar Chaudhary

**Affiliations:** Department of Chemistry, Mahendra Morang Adarsh Multiple Campus, Biratnagar, (Tribhuvan University) Nepal

**Keywords:** SEM-EDX, Anticorrosion activity, Surfactant, Ligand, Thermokinetics

## Abstract

This manuscript reports a dataset for the scanning electron microscopy with energy dispersive X-ray analysis (SEM-EDX), surfactant properties, thermokinetics, and corrosion inhibition activity of [[Co(HL)_2_.2H_2_O] Cl_2_.H_2_O]] (**1**) and [[Zn(HL)_2_.Cl] Cl.3H_2_O]] (**2**) complexes with surfactant-based Schiff base ligand (**HL**). It contains analyzed data related to thermokinetics, such as the activation energy (E*), entropy change (∆S*), enthalpy change (∆H*), and free energy change (∆G*) of **HL** and metal complexes. It also contains the SEM micrographs and EDX images of the studied ligand and metal complexes. A detailed analysis of the critical micelle concentration (CMC) data and figures illustrating the surfactant behavior of the synthesized complexes are presented in this article. The data for the corrosion inhibition activity of all synthesized compounds are also included. The dataset is related to the research article entitled “Bioinorganic interest on Co(II) and Zn(II) complexes of pyrrole-based surfactant ligand: Synthesis, characterization, and in silico-ADME study”.

**Specifications Table**

**Table:** 

Subject	Materials science

Specific subject area	Analytical Chemistry, Corrosion, and surface science
Type of data	Tables, Images, and Figures
How the data were acquired	Thermogravimetric analysis: Perkin Elmer STA6000 thermal analyzer under the N_2_ atmosphere. The slopes and intercepts of thermogravimetric analysis and differential thermal analysis (TGA/DTA) plots were analyzed to obtain thermokinetics data. A JEOL 6390 LA scanning electron microscope was used to generate SEM micrographs of the ligand and complexes and EDX images of complexes. Conductivity: An Auto Ranging digital conductivity TDS meter TCM 15+ was used to record conductivity data at 308 and 318 K temperatures. Corrosion inhibition: The weight loss method was used to evaluate corrosion inhibition on carbon steel coupons using a four-digit digital balance (Sartorius QUINTIX 224-1S analytical balance).
Data format	Raw and analyzed data
Description of data collection	Using Origin software, thermokinetic parameters were generated from TGA/DTA data. The conductivity data were processed in easy plot software to calculate CMC and Gibb's free energy of micellization. The SEM-EDX imaging was performed to analyze surface morphology and elemental composition. Corrosion inhibition data on carbon steel coupons were used to measure anticorrosion efficacy.
Data source location	Department of Chemistry, Mahendra Morang Adarsh Multiple Campus, Biratnagar, (Tribhuvan University), Nepal. SAIF, STIC Cochin, India.
Data accessibility	*Analyzed data and images are included with the article, and the raw data is deposited in the repository.* *Repository name: Mendeley Data* DOI:10.17632/99f4dxttm8.*2* https://data.mendeley.com/datasets/99f4dxttm8
Related research article	*J. Adhikari, A. Bhattarai, N. K. Chaudhary, Bioinorganic interest on Co(II) and Zn(II) complexes of pyrrole-based surfactant ligand: Synthesis, characterization, and in silico-ADME study, J. Mol. Struct., 1274 (2023), 134434*. https://doi.org/10.1016/j.molstruc.2022.134434

## Value of the Data

• Researchers in the area of material science may find the present data useful in investigating kinetic behavior and surfactant properties of compounds used in drug delivery systems.
• The SEM-EDX images are helpful in identifying morphological changes that occur during complexation of the ligand for the formation of metal complexes.
• Corrosion inhibition efficiency data collected for carbon steel (CS) coupons can be used to evaluate the efficacy of the inhibitor in other corrosive solutions (media).
• This result can be used to compare the thermal stability and decomposition rates of other surfactant-fabricated metal complexes.

## 1 Objective

The surface properties of the metal complexes make them particularly suitable for pharmaceutical applications. Several factors influence biochemical interactions with pathogens, including adsorption ability, thermokinetic stability, and surface morphology. The main goal of this study is to explore the data on how they selectively bind to specific regions of the pathogens by forming micellar aggregates at different temperatures based on degrees of micellization and free energy of micellization. The surface morphology and grain size of the complexes determine drug delivery effectiveness. Their anti-corrosion properties also make them ideal for a wide range of metal-based components as well as pharmaceutical applications. The datasets included in this manuscript are additional quantitative parameters that will add significant value to the complexes and support our previous publications [1,2].

## 2 Data Description

**Fig. 1:**
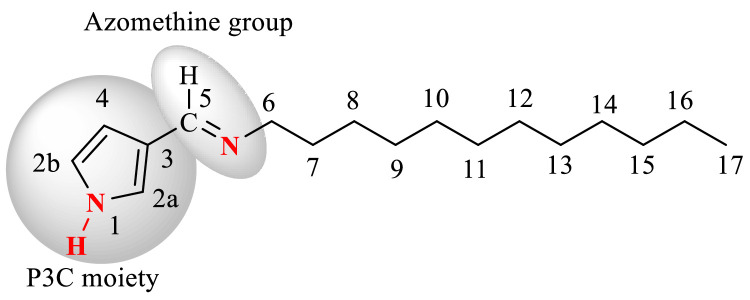
Structure of Schiff base ligand (HL).

In this study, we share SEM-EDX, thermokinetics, CMC, free energy of micellization, and anticorrosion activity data for [[Co(HL)_2_.2H_2_O] Cl_2_.H_2_O]] (**1**) and [[Zn(HL)_2_.Cl] Cl.3H_2_O]] (**2**) complexes with surfactant-based Schiff base ligand (**HL**) (Fig 1). The ligand was prepared by refluxing of a mixture of an ethanolic solution of pyrrole-3-carbaldehyde (**P3C**) and laurylamine (**LA**) in a 1:1 stoichiometric ratio.

**Fig. 2:**
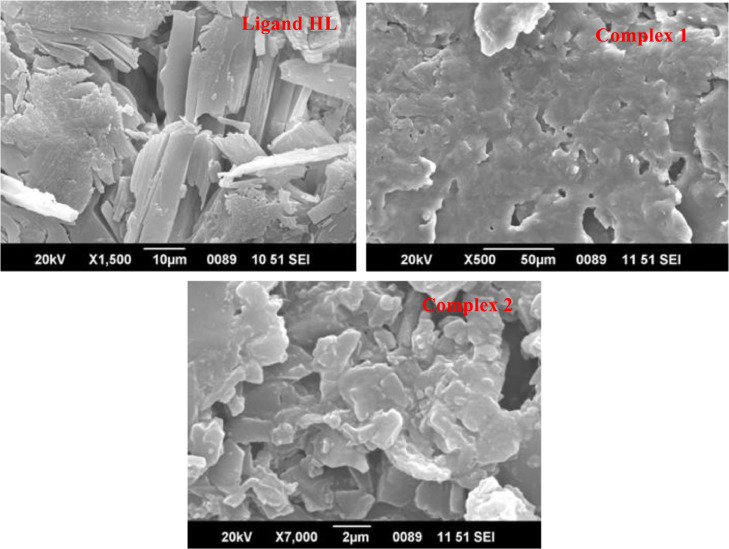
SEM micrograph of (a) Ligand (HL), (b) Complex 1, (c) Complex 2.

The SEM micrographs of ligand and complexes are presented in Fig. 2. The micrographs illustrate the variation in surface morphology for metal complexation of ligands with metal ions. The somewhat rod-shaped crystalline structure of the micrograph of **HL** has changed to the unevenly distributed morphological mass of the complexes after complexation.

Table 1 reports the elemental composition of the complexes detected from EDX analysis. It revealed the presence of various non-metal atoms, such as N, O, and Cl, and the respective metals. The EDX data indicated the expected elemental composition of the complexes as well, and the EDX images are shown in Mendeley Research data file [3].

**Table 1:** Elemental composition of complexes detected from EDX analysis.

Element	Complex **1**		Complex **2**	
	Weight (%)	Atomic (%)	Weight (%)	Atomic (%)
C	79.81	91.51	55.18	79.59
O	3.09	2.66	3.73	4.05
Cl	11.86	4.61	19.47	9.51
Co	5.2	1.21	-	-
Zn	-	-	19.19	5.08

The Coats-Redfern equation was used to calculate the thermodynamic and kinetic parameters of each decomposition step for the synthesized complexes. The results are presented in Table 2. A consecutive increase in the E* value in each decomposition step was observed. Table 2 showed negative ΔS*, positive ΔH*, and positive ΔG* values for all complexes [4], [5], [6]. Details regarding these parameters can be found in Section 3.2.

**Table 2:** Thermodynamic and kinetic parameters.

Complexes	Step	*r*	*A* (s^−1^)	*T_max_* (K)	E* (kJ/mol)	ΔS* (J/K.mol)	ΔH* (kJ/mol)	ΔG* (kJ/mol)
Complex 1	1	-0.99	6.32×10^10^	391.28	86.13	-40.40	82.88	98.68
	2	-0.98	8.09×10^10^	471.69	108.39	-39.89	104.46	123.28
	3	-0.98	3.85×10^10^	685.83	153.15	-49.19	147.45	181.19
Complex 2	1	-0.99	3.75×10^10^	374.06	82.89	- 44.35	79.78	96.36
	2	-0.99	9.78×10^9^	482.24	100.59	-57.65	96.59	124.39
	3	-0.99	4.29×10^8^	702.57	126.27	- 86.77	120.43	181.39
	4	-0.99	1.72×10^12^	907.61	226.83	-19.94	219.28	240.08

Here, r is Pearson's correlation coefficient, and A is Arrhenius pre-exponential factor.

**Fig. 3:**
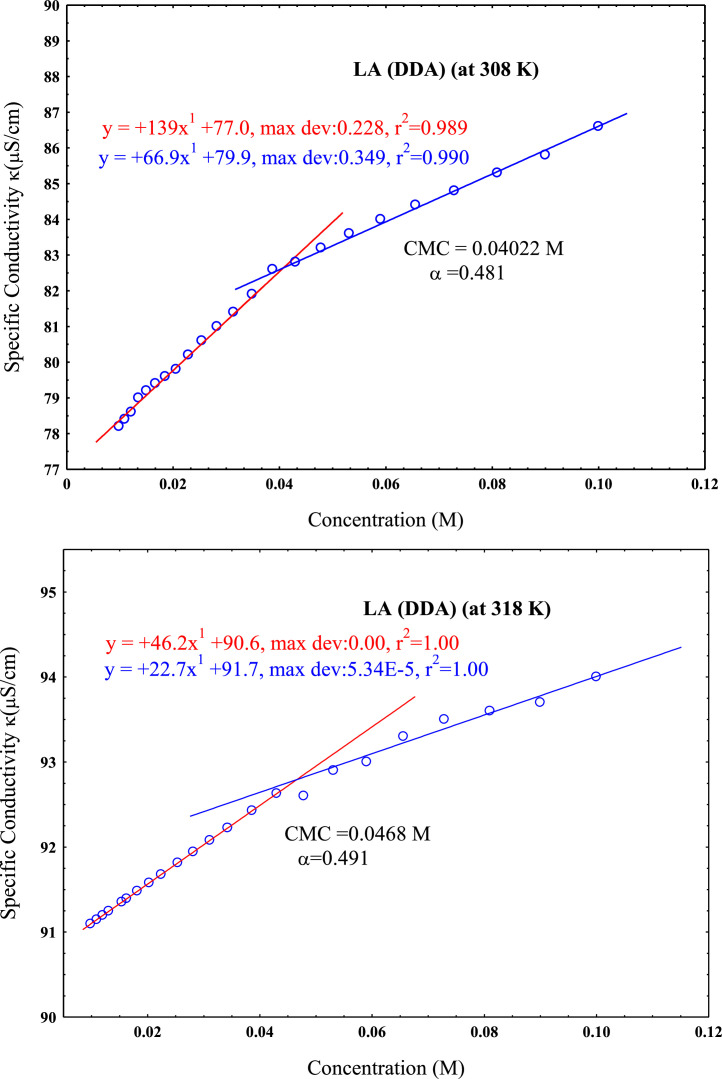
Plot of conductivity versus concentration of LA (DDA) at 308 K, and 318 K.

**Fig. 4:**
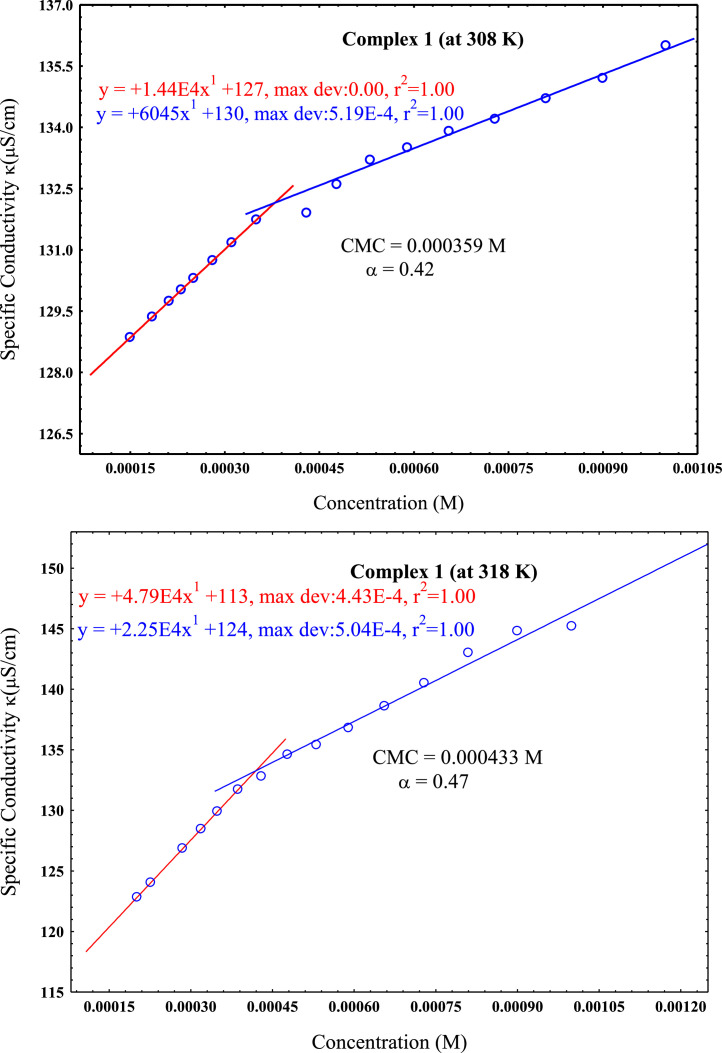
Plot of conductivity versus concentration of Complex 1 at 308 K, and 318 K.

**Fig. 5:**
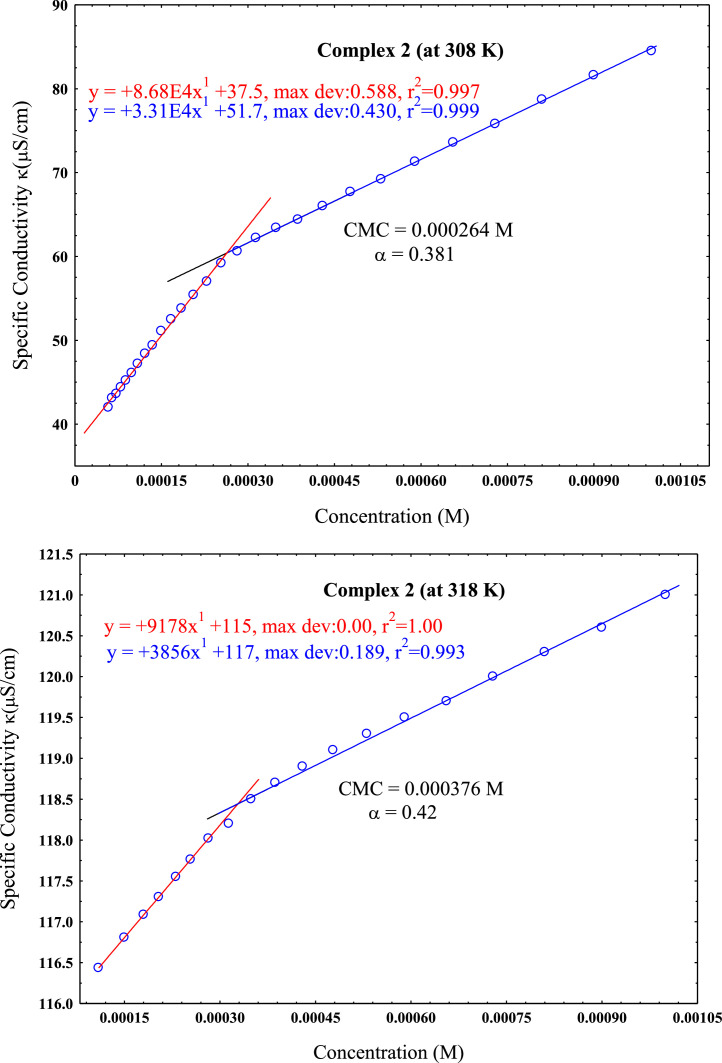
Plot of conductivity versus concentration of Complex 2 at 308 K, and 318 K.

The conductivity versus concentration plots of **LA**, complex **1** and complex **2** are shown in Fig. 3, Fig. 4, Fig. 5. The specific conductivity values varied before and after CMC. Owing to the formation of micelles with lower ionic mobility, the conductivity decreases after the CMC point [7]. Table 3 presents physicochemical data calculated from conductivity vs concentration plots. A higher CMC was reported for **LA** and complexes **1** and **2** at 318 K than at 308 K. A larger CMC value for **LA** (1.5 × 10^–2^ M) compared to complex **1** (3.21 × 10^−4^ M) and complex **2** (1.98 × 10^−4^ M) was also reported previously [1]. The Gibbs free energy of micellization $\left(\Delta G_{m}^{o}\right)$ was negative for all (Table 3) and became more negative from **LA** to complex **1** and then to complex **2** at a particular temperature, indicating spontaneity.

**Table 3:** Physicochemical parameters.

Compounds	T (K)	CMC (mol L^−1^)	$\Delta G_{m}^{o}$ (kJ/mol)	α = S_2_/S_1_	Std. Dev. in Linear Fit (SD)
**LA**	308	4.02×10^−2^	-12.50	0.48	0.1200
	318	4.68×10^−2^	-12.21	0.49	0.1703
**Complex 1**	308	3.59×10^−4^	-32.09	0.42	0.1738
	318	4.33×10^−4^	-31.33	0.47	0.7464
**Complex 2**	308	2.64×10^−4^	-34.16	0.38	0.2744
	318	3.76×10^−4^	-32.94	0.42	0.0561

Here S1and S2 represent premicellar and postmicellar slopes.

**Fig. 6:**
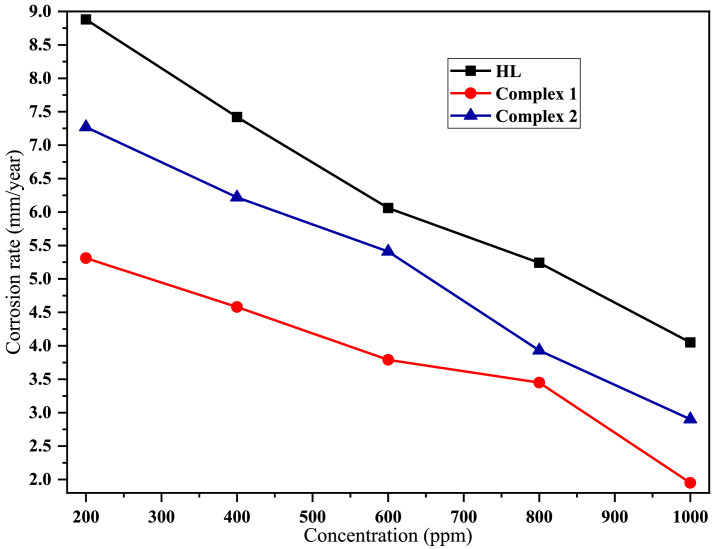
Variation of corrosion rate with concentration.

**Fig. 7:**
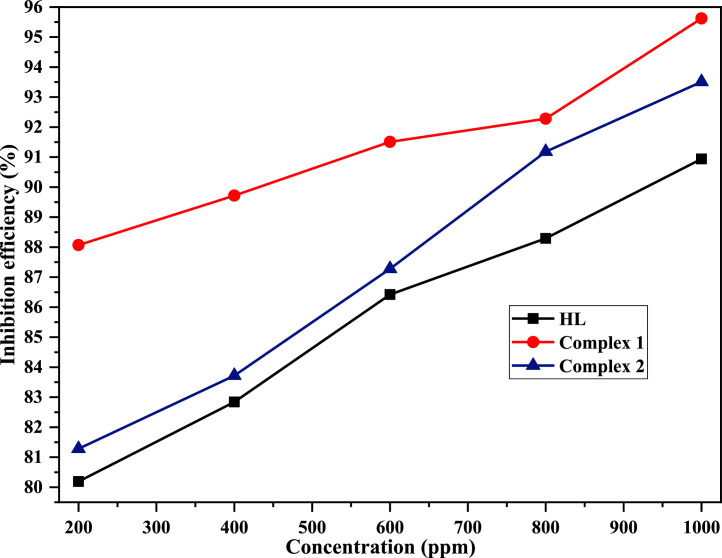
Variation of inhibition efficiency with concentration.

**Fig. 8:**
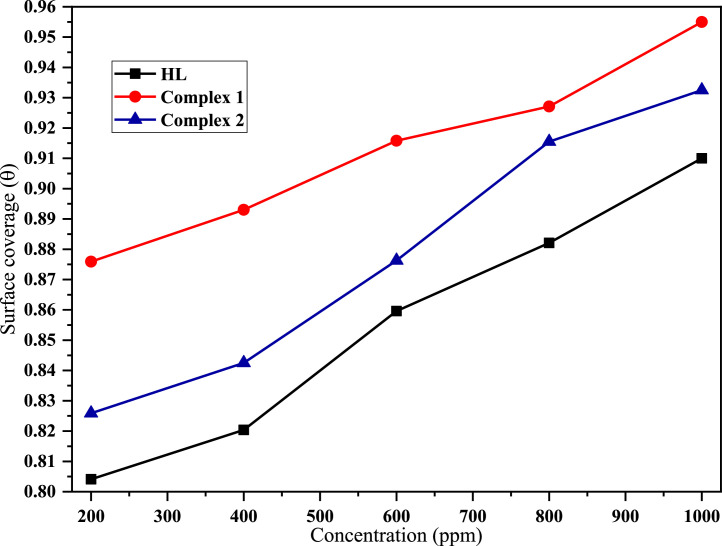
Surface coverage with concentration.

The anticorrosion activity was investigated using the weight-loss method, and the corrosion parameters are listed in Table 4. Table 5 presents the mass loss data for the carbon steel coupons during the experiments. Graphs showing the corrosion rate, inhibition efficiency, and surface coverage are shown in Fig. 6, Fig. 7, Fig. 8.

**Table 4:** Corrosion parameters.

Compound	Concentration (ppm)	Corrosion rate (mm per year)	Inhibition efficiency (η) (%)	Surface coverage (θ)
HL	200	8.88	80.19	0.8041
	400	7.42	82.84	0.8204
	600	6.06	86.42	0.8596
	800	5.24	88.29	0.8821
	1000	4.05	90.94	0.9100
Complex **1**	200	5.31	88.07	0.8759
	400	4.58	89.72	0.8930
	600	3.79	91.51	0.9158
	800	3.45	92.28	0.9271
	1000	1.95	95.62	0.9550
Complex **2**	200	7.27	81.28	0.8259
	400	6.22	83.72	0.8425
	600	5.41	87.28	0.8763
	800	3.93	91.18	0.9155
	1000	2.90	93.51	0.9325
**Control (1N HCl)**	44.68

**Table 5:** Mass loss of carbon steel coupons.

Compounds				
1N HCl (Control)	Concentration (ppm)	HL	Complex 1	Complex 2
Mass loss (gm)		Mass loss (gm)	Mass loss (gm)	Mass loss (gm)
0.089	200	0.0175	0.0110	0.0135
	400	0.0150	0.0095	0.012
	600	0.0125	0.0075	0.011
	800	0.0105	0.0065	0.0075
	1000	0.0080	0.0040	0.006

## 3 Experimental Design, Materials and Methods

### 3.1 SEM-EDX Analysis

SEM-EDX analysis was performed in JEOL 6390 LA scanning electron microscope with 3,00,000 X magnification, and accelerating voltage of 0.5-30 kV. EDX resolution was 136 eV under the detection area 30 mm^2^.

### 3.2 Thermogravimetric Analysis

TGA/DTA analysis was performed in Perkin Elmer STA6000 thermal analyzer with vertical type furnace under N_2_ atmosphere. The heating scan of the sample was done from 40 to 750°C at a linear heating rate of 10°C/min. The data were processed in Origin software to extract thermokinetics parameters. The parameters such as E*, ΔH*, ΔS*, and ΔG* of each decomposition step were evaluated using the Coast-Redfern equation (1) [8].

(1) $$\ln\left[-\frac{\ln\left(1-\alpha \right)}{T^{2}}\right]=ln\left[\frac{AR}{\beta E^{*}}\right]-\frac{E^{*}}{RT}$$

Where *α* is the fraction decomposed at time t, *β* denotes the linear heating rate, *A* denotes the Arrhenius pre-exponential factor, and *R* represents the general gas constant. A plot of the left-hand side against 1000/T of equation (1) gives a straight line whose slope (-E*/R) determines the activation energy (kJmol^−1^), and the intercept indicates the value of *A* in the s^−1^ unit. Using equations (2, 3, and 4), other thermodynamic parameters such as the ΔG*, ΔH*, and ΔS* were determined [6]. In equation (2), *k_B_* is Boltzmann constant and *h* is Plank's constant.

(2) $$\Delta S^{*}=Rln\left[\frac{Ah}{k_{B}T}\right]$$

(3) $$\Delta H^{*}=E^{*}-RT$$

(4) $$\Delta G^{*}=\Delta H^{*}-T\Delta S^{*}$$

### 3.3 Surfactant Activity Study

Conductivity measurements were performed to investigate the surfactant properties of the synthesized complexes. The study was conducted at temperatures of 308, and 318 K. The CMC was determined by plotting the specific conductivity (*κ*) against the concentration of the surfactant solution. The intersection of the two lines defines the CMC point, from which the degree of micellization (α) was calculated using the formula:

(5) $$\alpha ={S_{2}/S}_{1}$$

Similarly, Gibb's free energy of micellization was calculated from the formula:

(6) $$\Delta G_{m}^{o}=RT\left(2-\alpha \right)ln\;CMC$$

### 3.4 Corrosion Inhibition Activity Study

Carbon steel (CS) coupons cut to sizes of 2 cm × 2 cm × 0.07 cm were abraded with 80, 320, 600, 800, 1000, and 1200 grade emery (silicon carbide) papers. Stock solutions (1000 ppm) of **HL**, complex **1**, and complex **2** inhibitors were prepared in 100 mL of a 1.0 N HCl solution. The stock solution was diluted to obtain solutions with the desired concentrations of 800, 600, 400, and 200 ppm. Distilled water-washed, acetone-dried, and moisture-free abraded coupons were weighed and immersed in 25 mL of diluted solutions in crucibles with and without inhibitors. After 6 h of exposure, the coupons were removed and weighed as described above. Measurements were performed in triplicate to reduce errors. The size of each coupon was measured by using a digital screw gauge. The corrosion rate (CR) (mm/yr), inhibition efficiency (IE) percent (*η*), and surface coverage (θ) were calculated using the following equations (7-9) [9].

(7) $$\text{Corrosion}\;\text{rate},\mathrm{CR}=\frac{87600\times \Delta w}{d\times A\times t}$$

Where *∆w* represents the weight loss in grams, d is the density of the CS in grams per cc, A is the area of the CS coupons, and t represents immersion time in hours.

(8) $$\text{Inhibition}\;\text{efficiency}\;\%,\eta =\frac{\mathrm{CR}-CR^{\prime }}{\mathrm{CR}\;}\times 100\%$$

Here, CR and CR' denote the corrosion rates in the absence and presence of the inhibitors, respectively.

(9) $$\text{Surface}\;\text{coverage},\theta =\frac{W_{1}-W_{2}}{W_{1}}$$

Where, *w_1_* and *w_2_* represent the weight reduction in the absence and presence of the inhibitor, respectively.

## CRediT authorship contribution statement

**Janak Adhikari:** Methodology, Investigation, Data curation, Writing – original draft. **Ajaya Bhattarai:** Writing – review & editing. **Narendra Kumar Chaudhary:** Supervision, Conceptualization, Data curation, Software, Formal analysis, Writing – review & editing.

## Declaration of Competing Interest

The authors declare no competing financial interests or personal relationships that could influence the study reported in this article.

## Data Availability

SEM-EDX, thermokinetics, surfactant and corrosion inhibition activity of Co(II) and Zn(II) complexes of (E)-N-((1Hpyrrol-3yl)methylene)dodecan-1-amine Schiff base (Original data) (Mendeley Data).
